# Awareness of antimicrobial resistance and appropriate handling of antibiotics by the public in Saudi Arabia: A cross-sectional study using a quiz game

**DOI:** 10.1016/j.pecinn.2024.100318

**Published:** 2024-07-04

**Authors:** Waad M. Alzahrani, Lujain S. Alkliakh, Esraa B. Alwafai, Manal F. Madani, Nima L. Hersi, Eilaaf A. Shakir, Abrar K. Thabit

**Affiliations:** a Faculty of Pharmacy, King Abdulaziz University, Jeddah, Saudi Arabia; b Pharmacy Program, Batterjee Medical College, Jeddah, Saudi Arabia; c Pharmacy Practice Department, Faculty of Pharmacy, King Abdulaziz University, Jeddah, Saudi Arabia

**Keywords:** Antimicrobial resistance, Antibiotic, Public health, Awareness, Saudi Arabia

## Abstract

**Objective:**

Public awareness of antimicrobial resistance (AMR) is essential to tackling this issue. Studies in Saudi Arabia have demonstrated insufficient AMR knowledge among the public. We aimed to indirectly raise awareness while simultaneously assessing the public's knowledge. We also assessed the factors associated with optimal knowledge and antibiotic handling.

**Methods:**

We developed an online quiz game comprising 10 questions on AMR knowledge and antibiotic handling, recording each participant's score. We collected the responses from the Saudi public using a cross-sectional study design.

**Results:**

Of the 428 participants, 68.7% were females and 42.5% were aged between 41 and 65 years; 70.1% held undergraduate degrees. Only 17.8% had a health-related major. While 83.2% had heard of AMR, the median [interquartile range] AMR knowledge score was 6 [5–7] out of 10 points. Holding a health-related major and having prior knowledge of AMR were associated with higher scores (RR, 1.28 and 1.18; 95%CI, 1.13–1.44 and 1.03–1.35; *P* < 0.001 and *P* = 0.020, respectively).

**Conclusion:**

The Saudi public demonstrated average knowledge of AMR. We recommend awareness-raising campaigns about AMR targeting the public.

**Innovation:**

We utilized an innovative approach by distributing an online questionnaire as a quiz game to fulfill two purposes: the assessment of knowledge and awareness-raising about AMR.

## Highlights

• Public awareness of antimicrobial resistance (AMR) is essential to tackle the issue.
• We utilized a scored online quiz game to assess the knowledge of the public in Saudi Arabia on AMR.
• The public in Saudi Arabia demonstrated an average AMR knowledge.
• Holding a health-related major and having heard of AMR were significantly associated with higher scores.
• AMR awareness-raising campaigns are recommended to spread knowledge on AMR and improve antibiotic handling by the public.

## 1 Introduction

Microorganisms, particularly bacteria, constantly evolve and develop resistance when exposed to antibiotics, whether through human intake or from agricultural practices, resulting in the consumption of antibiotic-containing foods (such as meat, poultry, and vegetables) [1]. The overuse and misuse of antibiotics without clear indications have led to the emergence of antibiotic-resistant bacteria, posing a significant threat to public health [2]. Antimicrobial resistance (AMR) is a major global challenge that complicates the treatment of infections. The lack of public knowledge exacerbates this issue. The World Health Organization (WHO) emphasizes the importance of public education and awareness to mitigate this risk, notably through its annual World AMR Awareness Week that takes place in November of each year [3].

Saudi Arabia is one of the countries that suffer from an increasing level of AMR over the years based on data from various Saudi hospitals [4]. Prior to 2018, antibiotics in Saudi Arabia could be dispensed without a prescription, where people could simply obtain antibiotics from any community pharmacy even without a proper indication. Such an issue is assumed to have been a major factor contributing to the AMR issue in the country. In April 2018, the Saudi Ministry of Health issued a legislation prohibiting pharmacists from dispensing antibiotics without a prescription as part of the Executive Regulations of Health Practice Law [5]. The passing of this legislation was announced in various social media platforms accompanied by an awareness campaign on antibiotics and AMR catered to the public. Despite these efforts, the level of knowledge of the Saudi public on AMR remained below average based on results of a few studies that were conducted after the passing of this mandate [[6], [7], [8]]. Similar findings were also reported in studies from different countries around the Arab world several studies assessing knowledge of AMR and appropriate antibiotic handling have shown that a large population exhibits insufficient knowledge [9,10].

The previous survey studies that assessed the public's knowledge of AMR mostly utilized standard questionnaires. Therefore, to enhance the effectiveness of this type of cross-sectional method, our study aimed to raise awareness about the AMR threat and assess the public's knowledge of it and their attitudes toward infections and antibiotics. The second objective of the study was to evaluate the factors associated with optimal AMR knowledge and attitudes toward infections and antibiotic handling.

## 2 Methods

### 2.1 Study design and population

This was a cross-sectional study utilizing an online questionnaire distributed via different social media platforms, including WhatsApp, X (formerly Twitter), and Snapchat. The survey link was accessible and active for six days, with the responses collected between November 20 and November 26, 2023.

The eligible participants included adolescents and adults aged 14 years and older from the Saudi public. Ethical approval was granted by the Research Ethics Committee of the Faculty of Pharmacy at King Abdulaziz University.

### 2.2 Questionnaire

The questionnaire was presented in Arabic using Google Forms. A copy of the questionnaire translated to English as well as the original survey in Arabic are available in the supplementary materials. The questionnaire comprised 17 questions. The demographic section contained seven items related to participants' gender, age, education level, whether they held a major in health, whether they had chronic diseases, whether they had heard of AMR, and their source(s) of information about AMR, if applicable. The remaining 10 questions comprised the quiz game and were dedicated to assessing knowledge of AMR and attitudes toward the handling of antibiotics and the management of infections. These questions featured multiple-choice options with checkboxes, allowing the selection of a single or multiple answers (except for one yes/no question that had a yes/no option regarding the use of antibiotics to treat colds and rhinitis). Considering scoring, each correct response earned 1 point, while incorrect answers or correct answers combined with incorrect ones resulted in zero points. A score from 0 to 3 indicated poor knowledge, a score from 4 to 6 indicated an average knowledge, and a score ≥ 7 indicated a good knowledge. Upon submitting their answers, the participants could view their scores and the questions they answered correctly while seeing the correct answers to each question. However, the participants could not return to the survey page to correct their responses.

To avoid the duplication of responses, email addresses were anonymously collected from each participant, which were hidden from the study investigators to maintain the confidentiality of the participants. Prior to the wide distribution of the survey, it was first piloted with five participants, where issues related to questions' clarity were corrected according to the participants' and investigators' feedback to ensure that that the questions are understandable by the public. This was followed by a second pilot phase with another five participants to ensure the validity and test-retest reliability.

### 2.3 Statistical analysis

The data were analyzed descriptively, and categorical variables were presented as frequencies and percentages. To assess normality of distribution of the continuous data, the Shapiro-Wilk test was conducted, which indicated lack of normal distribution. Therefore, continuous data were presented as median and interquartile range (IQR). Cronbach's alpha was not calculated since the survey included multiple-choice questions rather than dichotomous and scaled questions. Poisson regression analysis was conducted to evaluate the association of different factors (independent variables) with higher scores on the quiz.

A minimum sample size of 385 participants was required to achieve a confidence level of 95% with a margin of error of 5% based on a population size of 36 million in Saudi Arabia. Statistical significance was established at a *P* < 0.05. Statistical analysis was carried out using SPSS version 28.0 (IBM Corp., Armonk, NY, USA).

## 3 Results

A total of 428 participants completed the questionnaire, predominantly females (68.7%) aged 41–65 years (42.5%), followed by those aged 26–40 years (34.3%). The majority held an undergraduate degree (70.1%), but only 17.8% had a health-related major. The primary source of knowledge was a healthcare provider (HCP; 43.7%), followed by social media (24.5%). Table 1 lists the remaining characteristics of the participants.

**Table 1:** Characteristics of participants (*n* = 428).

Characteristic	n (%)
Gender (Female)	294 (68.7)
Age (years)	
14–17	7 (1.6)
18–25	89 (20.8)
26–40	147 (34.3)
41–65	182 (42.5)
> 65	3 (0.7)
Education	
None	1 (0.2)
Elementary	2 (0.5)
Middle School	9 (2.1)
High School	76 (17.8)
Undergraduate	300 (70.1)
Postgraduate	40 (9.3)
Health major	76 (17.8)
Having a chronic disease	70 (16.4)
Heard of AMR	356 (83.2)
Source of information	
Education	15 (3.5)
Healthcare provider	187 (43.7)
Social media	105 (24.5)
Media: TV/Radio	30 (7.0)
Family/friends	63 (14.7)

AMR, antimicrobial resistance.

While 83.2% of the participants reported hearing about AMR, the median [IQR] score for appropriate AMR knowledge and antibiotic handling was 6 [5–7] out of 10 points, which indicated an overall average score. Table 2 shows the quiz questions and the proportion of participants who correctly answered each question. In terms of knowledge about antibiotics, 220 of 428 respondents (51%) demonstrated an understanding that antibiotics work specifically against bacteria. Furthermore, 46% (*n* = 197) of the participants were familiar with the term AMR. However, a relatively small proportion of participants (*n* = 81; 18.9%) were aware that antibiotics could not be dispensed without a prescription. The study revealed that 50.5% (*n* = 216) of the respondents sought medical attention when experiencing a sore throat. Although a limited number of participants (*n* = 71; 16.6%) correctly acknowledged that antibiotics are not effective in treating cold and rhinitis, a substantial 78.3% (*n* = 355) were aware of the type of infection that causes a cold. Furthermore, 71.7% (*n* = 307) demonstrated good knowledge regarding the importance of stopping antibiotic use according to the prescribed period by the doctor. Additionally, 64.3% (*n* = 275) reported taking their missed antibiotic doses as soon as possible upon remembering. Finally, more than one-third (*n* = 162; 37.9%) were knowledgeable about the side effects associated with unnecessary antibiotic consumption, such as diarrhea. The primary sources of information on AMR and antibiotics were HCPs (43.7%), followed by social media (24.5%).

**Table 2:** Number (percentage) of participants who selected the correct answers.

Question	Selected the correct answer only	Selected the correct answer plus other incorrect choices
1. What are antibiotics?	220 (51.4)	89 (20.8)
2. What is AMR?	197 (46)	230 (53.7)
3. Why are antibiotics not dispensed only without a prescription?	81 (18.9)	118 (27.6)
4. What do you do when you have a sore throat?	216 (50.5)	291 (68.0)
5. Do antibiotics treat cold and rhinitis?	71 (16.6)	NA⁎
6. What type of infection that causes cold?	355 (78.3)	50 (11.7)
7. When antibiotic should be stopped?	307 (71.7)	367 (85.7)
8. What to do when forgetting an antibiotic dose?	275 (64.3)	302 (70.6)
9. Consuming antibiotics without a need can result in side effects, such as…	162 (37.9)	266 (62.1)
10. How to protect yourself from an infection?	364 (85)	416 (97.2)
Score (median [IQR])	6 [5–7]	NA

AMR, antimicrobial resistance; IQR, interquartile range; NA, not applicable.

⁎ This question had two options only (yes or no).

The results of the logistic regression to analyze factors potentially associated with a high score are reported in Table 3. We found that holding a health-related major and having heard of AMR were significantly associated with higher scores (RR, 1.28 and 1.18; 95% CI, 1.13–1.44 and 1.03–1.35; *P* < 0.001 and *P* = 0.020, respectively). Gender, age, source of AMR knowledge, and educational level were not significant predictors of a sufficient knowledge level.

**Table 3:** Factors associated with higher total score.

Factor	RR	95% Confidence Interval	*P* value
Gender (Female)	0.98	0.90–1.07	0.687
Age (years)			
14–17	Ref	Ref	–
18–25	1.08	0.74–1.57	0.699
26–40	1.24	0.86–1.805	0.252
41–65	1.26	0.873–1.83	0.215
> 65	1.46	0.82–2.59	0.195
Education			
None	Ref	Ref	–
Elementary	0.87	0.26–2.96	0.825
Middle School	1.01	0.39–3.01	0.861
High School	1.44	0.53–3.86	0.474
Undergraduate	1.43	0.53–3.83	0.478
Postgraduate	1.62	0.60–4.36	0.342
Holding a health major	1.28	1.13–1.44	< 0.001
Having a chronic disease	0.98	0.87–1.09	0.672
Heard of AMR	1.18	1.03–1.35	0.020
Source of information			
Education	0.96	0.72–1.28	0.766
Healthcare provider	0.83	0.67–1.02	0.076
Social media	0.91	0.74–1.12	0.369
Media: TV/Radio	0.82	0.64–1.05	0.108
Family/friends	0.84	0.67–1.05	0.119

AMR, antimicrobial resistance.

## 4 Discussion and conclusion

### 4.1 Discussion

AMR poses a serious global public health threat that renders the treatment of infectious diseases more challenging. One effective way to tackle the AMR dilemma is to raise public awareness and provide education about AMR in accordance with recommendations made by the WHO [11]. In this study, we assessed the knowledge, understanding, and attitudes regarding AMR and antibiotic use in Saudi Arabia.

We found that the majority of the respondents (83%) had heard of AMR from HCPs (43.6%). Holding a health-related major and having prior knowledge of AMR were significantly associated with higher scores on AMR knowledge and appropriate antibiotic handling (RR, 1.28 and 1.18; 95% CI, 1.13–1.44 and 1.03–1.35; *P* < 0.001 and *P* = 0.020, respectively). These findings align with those of studies from Saudi Arabia, Europe, and Pakistan, which also reported that better AMR knowledge was significantly associated with being a practitioner in the medical field [6,8,12,13].

In our study, social media also played a role, being the second most common source of AMR knowledge, as reported by 24.5% of the participants. This observation is in line with the growing importance of social media as a source of health information. This was demonstrated in a study that evaluated the quality and reliability of Arabic AMR videos on YouTube, which found that most of these videos, whether from official or unofficial sources, have good overall quality and reliability; hence, the authors encouraged using such videos in AMR awareness campaigns [14]. Moreover, a systematic review by Almohammed et al. reported that 28% (*n* = 111 of 397) of participants in studies evaluating AMR knowledge in the Arab Gulf countries used social media as their source of information on AMR [10].

Our study demonstrated that the 41–65-year-old group (42.5%) had the highest scores in knowledge and optimal attitudes toward antibiotic use. A study by Alduhaimi et al. from Saudi Arabia found a trend toward better knowledge with older age [6]. Conversely, another study from Saudi Arabia observed that individuals aged 18–24 years had better knowledge about antibiotics than other age groups [7]. Better knowledge of AMR and attitude toward antibiotics with older age could potentially be explained by the greater life experience of older individuals, including more frequent exposure to HCPs and social media. The latter was also the medium through which the Saudi Ministry of Health carries out awareness campaigns, especially after passing the legislation that prohibits the dispensing of antibiotics without a prescription [5]. As this is only a cross-sectional study and does not measure that change in knowledge over time, it could be possible that the knowledge of the younger generation could improve with additional exposure to educational materials on social media. For instance, the study by Bajaba et al. found that the number of Arabic videos on AMR available on YouTube has increased over the years from 2014 to 2022 [14]; thus, the older generation may have been exposed to such resources (in addition to learning from their HCPs) compared with the younger generation who may have been young to understand such information when they were published.

Although two studies from Saudi Arabia found that the female gender was significantly associated with better AMR knowledge [6,7], our study, along with another from Saudi Arabia, did not find a correlation between gender and AMR awareness [8]. In fact, the substantial representation of females in our study, accounting for more than two-thirds (68.7%), could potentially explain the below optimal overall quiz score.

A previous report from Saudi Arabia that assessed the knowledge, attitudes, and practices regarding the use of antibiotics showed that almost half of the respondents (51.1%) belive that antibiotics could be used to treat viral infections, while 49.5% thought they could be used for colds and coughs [7]. Similarly, 51% of the respondents to our questionnaire demonstrated an understanding that antibiotics are effective specifically against bacteria, and 78.3% of the participants correctly identified viruses as the cause of colds. While this appears encouraging, only a small number of participants (16.6%) believed that antibiotics could be used to treat colds.

Appropriate handling of antibiotics was assessed in a series of questions, one of which concerned when to stop the antibiotic course. Notably, most participants (71.7%) answered that antibiotics should be stopped only after completing the prescribed duration according to the doctor's instructions. This large percentage of correct answers demonstrates responsible antibiotic use by acknowledging the importance of finishing prescriptions even when symptoms improve. Nevertheless, a minority of participants (8.18%) selected the option stating that antibiotics can be stopped if symptoms improve. In contrast, the Saudi studies by Shatla et al. and Alarni et al. found that 40% and 37% of respondents, respectively, would stop the antibiotic course when they started to feel better [7]. Our study also highlighted another positive aspect of antibiotic administration, where 64.3% of the respondents indicated that a missed antibiotic dose should be taken as soon as it is remembered rather than skipped, which was chosen by 34.3% of the participants. Correctly answering this question indicates awareness of proper actions for missed doses and promotes adherence to medication regimens. This knowledge ensures optimal antibiotic use and reduces the risk of treatment failure [15]. Similarly, understanding of the importance of hygiene and self-protection was shown by the high percentage of participants (85%) who correctly answered the question “How to protect yourself from an infection?,” which included hand washing, the use of sanitizers, and keeping a safe distance. However, a total of 416 (97.2%) participants selected this choice along with incorrect ones (such as consuming medicinal herbs [12.1%] and taking antibiotics everyday [3.3%]). It is assumed, however, that the reason for selecting the correct answer to this question is the enhanced public awareness regarding self-protection from respiratory infections that spread during the recent COVID-19 pandemic [16].

Finally, the study found that about half of the respondents (50.5%) mentioned seeking medical attention when experiencing a sore throat versus 7.5% who answered taking an antibiotic that was previously prescribed for the same condition. Such results suggest that some of the public is educated about the appropriate timing to seek medical advice and that antibiotics should not be used for self-limiting viral illnesses, such as colds and rhinitis.

Despite the valuable insights of our study, it is important to consider some of its limitations. First, the use of the Arabic language in the questionnaire excluded non-Arabic speakers among non-Saudi citizens in Saudi Arabia. Second, the predominance of female and older adult respondents may represent a narrow demographic, raising concerns about the inclusivity of the study's findings. Finally, its cross-sectional design restricts our understanding of how awareness and attitudes might change over time.

### 4.2 Innovation

While some previous studies from Saudi Arabia have assessed knowledge on AMR and antibiotic handling, this is the first study to utilize an innovative approach in the form of an online questionnaire that serves two purposes: assessment of knowledge and attitudes as well as raising awareness and enhancing knowledge on AMR. This was accomplished by creating the questionnaire as a scored quiz game, where the participants were able to see their scores and the correct answers to improve their knowledge of AMR and the appropriate handling of antibiotics and respiratory viral infections.

### 4.3 Conclusion

The public in Saudi Arabia demonstrated an average knowledge of AMR. Holding a health-related degree and having heard about AMR (mostly from an HCP) were significantly associated with better knowledge and practices in handling antibiotics. The results of the present study recommend the development and implementation of educational and awareness-raising campaigns about AMR for the general population, targeting specific education about AMR to groups with low levels of knowledge, such as those with no health-related education or those who have not heard of AMR. Additionally, HCPs are encouraged to continue educating their patients and the public about AMR in their practice areas and on social media to ensure better management of infections and antibiotics by the public.

## Funding

None.

## CRediT authorship contribution statement

**Waad M. Alzahrani:** Writing – original draft, Investigation, Conceptualization. **Lujain S. Alkliakh:** Writing – original draft, Investigation, Data curation. **Esraa B. Alwafai:** Writing – original draft, Investigation, Data curation. **Manal F. Madani:** Writing – original draft, Methodology, Investigation. **Nima L. Hersi:** Writing – original draft, Investigation. **Eilaaf A. Shakir:** Writing – original draft, Investigation. **Abrar K. Thabit:** Writing – review & editing, Writing – original draft, Supervision, Project administration, Formal analysis.

## Declaration of Competing Interest

The authors report no conflicts of interest, financial or otherwise, related to this study or submission.

## Appendix A Supplementary data

Supplementary material Image 1
