# Supplementary material for: Awareness of antimicrobial resistance and appropriate handling of antibiotics by the public in Saudi Arabia: A cross-sectional study using a quiz game
Source: PEC Innov. 2024 Jul 4;5:100318. doi: 10.1016/j.pecinn.2024.100318 (PMC11284676; doi:10.1016/j.pecinn.2024.100318)
Supplement: Supplementary file 1 — Supplementary material [file mmc1.docx]

**Supplementary table.** Questionnaire and quiz game questions

| **Question** | **Correct answer** |
| --- | --- |
| **What are antibiotics?** |  |
| Medicines that work against bacteria | ✓ |
| Medicines that work against viruses |  |
| Medicines that work against fungi |  |
| **What is AMR?** |  |
| Bacteria become aggressive and difficult to treat | ✓ |
| Increases antibiotic efficacy to kill the bacteria |  |
| Using antibiotic for a longer period |  |
| Resistance against all infections |  |
| **Why are antibiotics not dispensed without a prescription?** |  |
| Using antibiotics without a prescription increases AMR | ✓ |
| They are considered controlled drugs |  |
| To treat the disease with an appropriate medication |  |
| They affect the digestive system, such as nausea and vomiting |  |
| **What do you do when you have sore throat?** |  |
| Go to the doctor/seek medical attention | ✓ |
| Take an antibiotic that was previously prescribed to treat the same problem |  |
| Doing nothing |  |
| Take vitamin C |  |
| **Do antibiotics treat cold and rhinitis?** |  |
| Yes |  |
| No | ✓ |
| **What type of microbe causes cold?** |  |
| Cold is caused by viruses | ✓ |
| Cold is caused by bacteria |  |
| Cold is caused by fungi |  |
| **When should the antibiotic be stopped?** |  |
| When I feel better |  |
| When side effects appear |  |
| According to the period determined by the doctor | ✓ |
| Five days after the first dose of the drug |  |
| **What to do when forgetting an antibiotic dose?** |  |
| Take it when you remember as soon as possible | ✓ |
| Skip the dose and take it on the next day at the same time |  |
| Take two doses the next day |  |
| Stop taking the antibiotic |  |
| **Consuming antibiotics without a need can result in side effects, such as …** |  |
| Diarrhea | ✓ |
| Cough |  |
| Neuropathy |  |
| Dizziness and headache |  |
| **How to protect yourself from an infection?** |  |
| Hand wash, use sanitizer, and keep a safe distance | ✓ |
| Taking antibiotic everyday |  |
| Taking herbs/herbal products |  |

AMR, antimicrobial resistance
